# Subcellular western blotting of single cells

**DOI:** 10.1038/micronano.2016.79

**Published:** 2017-02-13

**Authors:** Kevin A. Yamauchi, Amy E. Herr

**Affiliations:** 1 Department of Bioengineering, University of California, Berkeley, CA 94720, USA; 2 The UC Berkeley—UCSF Graduate Program in Bioengineering, University of California, Berkeley, CA 94720, USA

**Keywords:** cytometry, microfluidic design, proteomics, single-cell analysis

## Abstract

Although immunoassays are the *de facto* standard for determining subcellular protein localization in individual cells, antibody probe cross-reactivity and fixation artifacts remain confounding factors. To enhance selectivity while providing single-cell resolution, we introduce a subcellular western blotting technique capable of separately assaying proteins in the 14 pL cytoplasm and 2 pL nucleus of individual cells. To confer precision fluidic control, we describe a passive multilayer microdevice that leverages the rapid transport times afforded by miniaturization. After isolating single cells in microwells, we apply single-cell differential detergent fractionation to lyse and western blot the cytoplasmic lysate, whereas the nucleus remains intact in the microwell. Subsequently, we lyse the intact nucleus and western blot the nuclear lysate. To index each protein analysis to the originating subcellular compartment, we utilize bi-directional electrophoresis, a multidimensional separation that assays the lysate from each compartment in a distinct region of the separation axis. Single-cell bi-directional electrophoresis eliminates the need for semi-subjective image segmentation algorithms required in immunocytochemistry. The subcellular, single-cell western blot is demonstrated for six targets per cell, and successfully localizes spliceosome-associated proteins solubilized from large protein and RNA complexes, even for closely sized proteins (a 7 kDa difference). Measurement of NF-κB translocation dynamics in unfixed cells at 15-min intervals demonstrates reduced technical variance compared with immunofluorescence. This chemical cytometry assay directly measures the nucleocytoplasmic protein distribution in individual unfixed cells, thus providing insight into protein signaling in heterogeneous cell populations.

## Introduction

Cellular characteristics can vary widely among a population of cells^1,2^. Among such characteristics, the subcellular location of a protein is inexorably linked to its function. In a canonical example, transcription factors can be inactive while in the cytoplasm but active once localized to the nucleus where these proteins then regulate transcription^3,4^. As such, simultaneously ascertaining protein identity and subcellular location yields insight into function and signaling state.

Transfection of fluorescently labeled proteins combined with fluorescence microscopy reports protein dynamics in single cells with high temporal resolution. For example, fluorescent proteins fused to the transcription factor NF-κB have been used to study the dynamics of the translocation of NF-κB from the cytoplasm to the nucleus in response to biochemical stimulus with ~5 min temporal resolution^5,6^. More recently, embryonic stem cells with fluorescently tagged Sox2 were imaged to quantify transcription factor-binding kinetics with residence times <1 s (Ref. 7). While enabling the measurement of translocation dynamics of proteins with high temporal resolution, fluorescent fusion proteins inherently require transfection of the cells of interest and thus do not allow for the analysis of unaltered, endogenous proteins, which is the focus of the present study.

The *de facto* standard for determining subcellular location of unmodified endogenous proteins in single cells is immunocytochemistry (ICC; or immunofluorescence, see Supplementary Table S1 for definitions of abbreviations used in the main text). Although broadly useful, quantitative ICC is fraught with challenges. Nonspecific background signal is problematic and arises from numerous sources, including antibody cross-reactivity and the fixation method^8,9^. To demarcate individual cells in micrographs, image analysis algorithms are employed but yield variable results when cell morphologies are diverse and the borders between cells are low-contrast^10,11^. Enhanced selectivity and throughput would bolster our ability to determine protein subcellular location in individual cells.

To enhance selectivity, western blotting combined with differential detergent fractionation (DDF) has been a mainstay^12^. The selectivity of western blots exceeds that of simple immunoassays by correlating the molecular mass (determined by an upstream electrophoretic separation) with a downstream immunoassay. To report subcellular localization, DDF western blotting uses a pair of specialized cell lysis buffers and two conventional slab-gel western blotting systems. The first lysis buffer lyses only the cytoplasmic compartment of the cells, intact nuclei are fractionated out, and then the pooled cytoplasm is assayed by western blotting. The second lysis buffer lyses the pooled nuclei and the resulting lysate is then subjected to a separate slab-gel western blot^13,14^. Although more selective than ICC, DDF western blotting lacks the detection sensitivity needed for single-cell resolution.

Single-cell protein analyses have benefited from microfluidic tools^15,16^. A glass capillary interfaced to an individual cell makes capillary electrophoresis separations possible^17–20^, with these ‘chemical cytometry’ approaches primarily focused on metabolomics and enzyme-based reaction monitoring. Electrophoretic analysis of whole cells has benefited from microfluidic systems that locate and lyse each cell at the head of a separation channel^20,21^. Microchip electrophoresis has been used to count low-copy number proteins^22^ and to measure cytoplasmic RNA and genomic DNA from single cells, both with a throughput of ~10 cells per experimental group^23^. To ensure adequate population sampling of tens to thousands of whole cells, single-cell lysate separations have been reported by forming cell-isolation microwells directly in separation media^24–29^.

To map the cytoplasmic protein profile to the nuclear protein profile in each of hundreds of individual cells, we introduce a subcellular single-cell western blot assay ((sc)^2^WB). Using a multilayered microfluidic device and an optimized DDF buffer system, we sequentially lyse and western blot the cytoplasm and then the nucleus of hundreds of individual mammalian cells. Lysis reagents are diffusively delivered from a lid layer to individual cells isolated in microwells, thus precisely controlling the serial application of reagents. In the polyacrylamide base layer, we spatially segregate analysis of each compartment to a distinct region of the separation axis in a new bi-directional electrophoresis format. The three-dimensional device and multistage assay are designed for straightforward operation in well-equipped life science laboratories.

## Materials and methods

### Chemicals

Hoechst 33342 (B2261), digitonin (D141), Triton X-100 (100×), sodium dodecyl sulfate (SDS, L4509), sodium deoxycholate (D6750), lipopolysaccharides (LPS) from *Escherichia* *coli* (L4524), tetramethylethylenediamine (TEMED, T9281), ammonium persulfate (APS, A3678), β-mercaptoethanol (M3148), and 30%T, 3.3%C acrylamide/bis-acrylamide (29:1; A3574) were purchased from Sigma-Aldrich (St Louis, MO, USA). In addition, 10× tris-glycine electrophoresis buffer (25 mM Tris, 192 mM glycine, pH 8.3 at 1×) was purchased from Bio-Rad (Hercules, CA, USA) and 20× Tris-buffered saline with Tween 20 (sc-362196) was purchased from Santa Cruz Biotechnology (Dallas, TX, USA). Deionized water (18.2 MΩ) water was obtained from a Millipore Ultrapure water purification system (Billerica, MA, USA). N-[3-[(3-benzoylphenyl)formamido]propyl] meth-acrylamide (BPMAC) was synthesized by Pharm-Agra Laboratories (Brevard, NC, USA)^25,26^. Paraformaldehyde (157-4) was obtained from Electron Microscopy Services (Hatfield, PA, USA).

### Antibodies

Antibodies include rabbit anti-turboGFP (1:35, PA5-22688, Pierce Antibody Products, Waltham, MA, USA, with AlexaFluor 647-conjugated anti-rabbit secondary antibody), rabbit anti-β-tubulin (1:10, ab6046, Abcam, Cambridge, MA, USA, with AlexaFluor 647-conjugated anti-rabbit secondary antibody), rabbit anti-SFPQ (1:10, ab38148, Abcam, with AlexaFluor 647-conjugated anti-rabbit secondary antibody), mouse anti-PTBP1 (1:10, H00005725-M01, Abnova, Taipei City, Taiwan, China, with AlexaFluor 555-conjugated anti-mouse secondary antibody), rabbit anti-calnexin (1:10, ADI-SPA-865, Enzo, Farmingdale, NY, USA, with AlexaFluor 647-conjugated anti-rabbit secondary antibody), mouse anti-GRP-75 (1:10, ab82591, Abcam, with AlexaFluor 555-conjugated anti-mouse secondary antibody), and rabbit NF-κB (1:30, 8242, Cell Signaling, Danvers, MA, USA, AlexaFluor 647-conjugated anti-rabbit secondary antibody).

### Cell culture

U373 MG human glioblastoma cells were obtained from the UC Berkeley Tissue Culture Facility via the American Type Culture Collection. Green fluorescent protein (GFP)-expressing U373 cells were stably transduced with TurboGFP via lentiviral infection (multiplicity of infection=10). The U373 MG cells have been discovered to share a common origin with the U251 human glioblastoma cell line. However, the cells have since diverged into distinct karyotypes^30^. Mouse embryonic fibroblasts expressing mitochondria-targeted enhanced GFP (EGFP)^31^ were gifted by Dr. Suzanne Wolff and Dr. Brendan Battersby.

All cells were cultured in high-glucose Dulbecco’s modified eagle medium (DMEM) (11965, Life Technologies, Carlsbad, CA, USA) supplemented with 1× MEM nonessential amino acids (11140050, Life Technologies), 100 U mL^−1^ penicillin-streptomycin (15140-122, Life Technologies), 1 mM sodium pyruvate (11360-070), and 10% fetal bovine serum (JR Scientific, Woodland, CA, USA) in an incubator at 37 °C with humidified 5% CO_2_ air.

### SU8 wafer and (sc)^2^WB fabrication

The SU8 wafers and scWB devices were fabricated as previously reported^25,26^. The microwells were 32 μm in diameter and 40 μm deep. The well spacing was 1 mm along the separation axis and 0.25 mm transverse to the separation axis. The polyacrylamide gel precursor (8%T acrylamide/bis-acrylamide, 3 mM BPMAC) was polymerized with 0.08% APS and 0.08% TEMED. The polyacrylamide lids were fabricated using previously reported photopatterning methods^32^. The precursor (15% 29:1 acrylamide/bis-acrylamide, 1% VA-086 in water) was polymerized for 45 s at 20 mW cm^−^^2^. The lids measured 50×70×0.5 mm.

### Hydrogel lid fabrication

The polyacrylamide lids were fabricated using previously reported photopatterning methods^32^. Briefly, the polyacrylamide precursor (15% 29:1 acrylamide/bis-acrylamide, 1% VA-086 in water) was introduced between two hydrophobic glass plates (coated with Gel Slick) separated by 500 μm tall spacers. The glass plates were then placed on top of a photomask containing the pattern for the lids (rectangle measuring 50×70 mm), and the polyacrylamide was polymerized with ultraviolet light (*λ*=365 nm) for 45 s at 20 mW cm^−^^2^. The completed lids measured 50×70×0.5 mm. Following fabrication, the lids were soaked in the appropriate buffer for 1 h.

### (sc)^2^WB operation

First, cells suspended in phosphate-buffered saline (PBS; 500 000 cells per mL) are pipetted on top of the base layer of the (sc)^2^WB. The cells are allowed to settle into the microwells for 10 min via sedimentation at 4 °C. Next, the excess cell suspension is washed off of the surface of the base layer of the (sc)^2^WB with PBS, leaving only the cells in the microwells. The PBS is exchanged out of the base layer by incubating in 1× Tris-glycine for 20 s. The cell-containing base layer is transferred to the electrophoresis chamber. The lid containing the cytoplasm-specific buffer (Supplementary Table S2) is placed on top of the base layer, initiating lysis. After the completion of lysis (Supplementary Table S3), the electric field is activated (40 V cm^−1^) and polyacrylamide gel electrophoresis (PAGE) is performed on the cytoplasmic fraction (Supplementary Table S3). Immediately following the cytoplasmic PAGE, the proteins are immobilized by exciting the benzophenone in the polyacrylamide gel with ultraviolet light for 45 s. Next, the lid containing the cytoplasm-specific buffer is removed and replaced with the lid containing the nucleus-specific buffer (Supplementary Table S2), initiating lysis of the intact nuclei retained in the microwells. After completion of the nuclear lysis, PAGE on the nuclear fraction is performed by applying an electric field in the opposite polarity of the cytoplasmic PAGE step (Supplementary Table S3). Following the completion of the nuclear PAGE step, the nuclear proteins are immobilized by exposing the (sc)^2^WB device to ultraviolet light for 45 s. Finally, the immobilized proteins are immunoprobed as previously described^25^.

### (sc)^2^WB quantification

Fluorescence was measured using a Genepix 4300A (Molecular Devices, Sunnyvale, CA, USA) and appropriate laser and filter sets. Image processing was performed using a custom MATLAB script. Briefly, the image was segmented in separation lanes. Gaussian distributions were fit to the protein bands. Signal-to-noise ratio (SNR)>3 and *R*^2^>0.7 were used as thresholds for peak calling. The peak widths for the area under the curve analysis were 4*σ*.

### Immunocytochemistry

The cells were settled on glass coverslips at a density of 5×10^5^ cells per cm^2^ and then were cultured and fixed with 4 °C paraformaldehyde for 15 min. The fixed cells were washed three times (5 minutes each) in PBS and then were blocked and permeabilized in staining buffer (5% donkey serum, 0.3% Triton X-100, in TBST). The cells were incubated with primary antibody (1:200, anti-NF-κB) diluted in staining buffer for 12 h at 4 °C and then were washed three times (5 minutes each) in staining buffer. Cells were incubated in secondary antibody (1:300, AF647-labeled anti-rabbit) for 1 h at 20 °C, washed three times (5 minutes each) and counterstained with Hoechst 33342 (1 μg mL^−1^). The cells were imaged as described below with fluorescence quantified using CellProfiler^11^.

### Fluorescence imaging

Stained cells were imaged with an Olympus IX-71 inverted fluorescence microscope (San Jose, CA, USA) equipped with an EMCCD camera iXon3 (Andor, Concord, MA, USA) and an X-Cite Exacte mercury arc lamp (Excelitas, Waltham, MA, USA) illumination source coupled to an automated shutter and attenuation system. All ICC imaging were performed with an Olympus UPlanFl 20× (numerical aperture (NA) 0.5) objective, and all cell lysis imaging were performed with an Olympus UPlanFl 20× (NA 0.5) objective.

## Results and Discussion

### Fundamental considerations for the (sc)^2^WB assay and device designs

**Figure 1:**
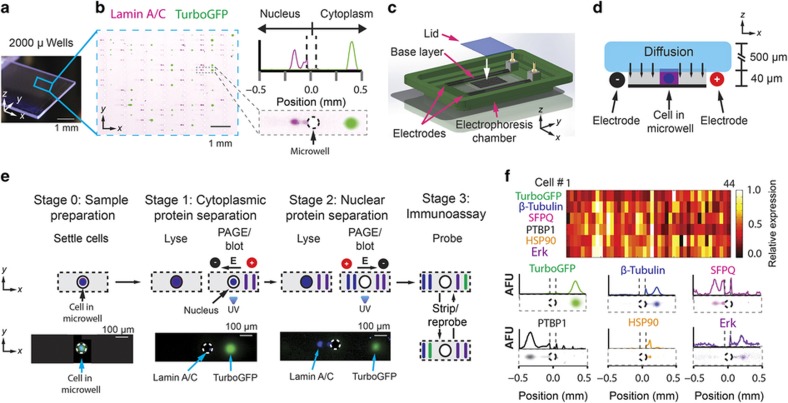
Microfluidic subcellular western blotting reports protein localization to the cytoplasmic or nuclear compartment of single cells. (**a**) Photograph of the base layer and microwell array of the (sc)^2^WB device, with (**b**) insets showing the fluorescence micrograph of the subcellular western blot array (56 U373 cells) for lamin A/C (magenta) and TurboGFP (green) and for a single U373 cell with a companion intensity profile plot. (**c**) Rendering of the assembled (sc)^2^WB device. (**d**) Schematic cross-section in the *x–z* plane of (**c**). When placed atop the base layer, the 500 μm-thick hydrogel lid simultaneously delivers the lysis reagents via diffusion and electrically addresses the base layer for rapid transition between the lysis and electrophoresis stages. (**e**) Schematic of the (sc)^2^WB workflow: (Stage 0) Settle single cells into microwells via sedimentation; (Stage 1) cytoplasm-specific lysis buffer is diffusively applied from the lid, PAGE is performed on solubilized cytoplasmic proteins along the separation axis to the ‘east’ of the microwell, and the cytoplasmic proteins are photo-immobilized to the gel; (Stage 2) nucleus-specific lysis buffer is diffusively applied from the lid, PAGE is performed on solubilized nuclear proteins along the separation axis to the ‘west’ of the microwell, and nuclear proteins are photo-immobilized to the gel; (Stage 3) in-gel immunoprobing and image fluorescence are performed. At bottom: (Step 0) false-color fluorescence micrographs of an intact cell in a microwell; (Step 1) PAGE of cytoplasmic GFP (*E*=40 V cm^−1^; Δ*t*=10 s) with the nucleus retained in the microwell; (Step 2) western blotting after bi-directional PAGE with cytoplasmic protein ‘east’ and nuclear protein ‘west’ of the microwell. The microwells are encircled with a white dashed line for clarity; TurboGFP (green) and Hoechst DNA stain (blue). (**f**) Stripping and reprobing for the expression and localization of six protein targets from one mammalian cell. The relative expression (AUC/AUC_max_) is reported for *n*=44 U373 cells. GFP, green fluorescent protein; PAGE, polyacrylamide gel electrophoresis.

The subcellular single-cell western blot assay or (sc)^2^WB isolates single cells in microwells and uses microfluidic control of DDF buffer pairs to lyse, solubilize, and electrophoretically analyze proteins from the cytoplasm and subsequently the nucleus of each isolated cell (Figure 1). Importantly, both the cytoplasmic western blot assay and the nuclear western blot assay are indexed to the originating microwell, thus allowing direct correlation between the cytoplasmic and nuclear protein profiles of each cell analyzed. The indexing is achieved by ‘bi-directional’ western blotting. In bi-directional western blotting, each interspersed PAGE assay is performed along the same separation axis; however, the protein solubilized from each subcellular compartment is electrophoresed in the opposite direction (that is, ‘east’ of the originating microwell for the cytoplasmic lysate and ‘west’ for the nuclear lysate).

Thus, the (sc)^2^WB consists of three stages (Figure 1). Stage 1, cytoplasmic protein separation: chemical lysis of the cytoplasm only, PAGE of solubilized cytoplasmic proteins while each intact nucleus is retained in the microwell, and blotting (photoimmobilization) of separated proteins to the hydrogel via ultraviolet (UV) activation of the photoactive gel. Stage 2, nuclear protein separation: chemical lysis of the intact nuclei, PAGE of solubilized nuclear proteins, and UV-based blotting of proteins to the hydrogel. And Stage 3, probing of both protein separations: probing and imaging of the immobilized proteins from each subcellular compartment with fluorescently labeled antibodies. Throughput of the microwell array can reach up to ~7000 cells per device^25^. However, the number of microwells with single-cell occupancy is cell-type-dependent (for example, depending on the density and morphology)^25,26^. For the cells studied here, a microwell occupancy of one was achieved for ~100 s of cells per device.

The small length scales of both the sample (cells) and device features make diffusion a dominant mass transport mechanism. While the protein concentrations are high within an intact, whole cell (nM to μM)^33^, the small dimensions of the subcellular compartments (*l*_nuc_~15 μm; *l*_cyt_~30 μm) lead to rapid dilution by diffusion after lysis (Supplementary Figure S1). To estimate the dilution effects in the (sc)^2^WB, we consider lysis of GFP from a single cell (molecular mass: 27 kDa; *D*=88 μm^2^ s^−1^)^25^. Using a diffusion timescale of *τ*=*x*^2^⁄2*D*, where *x* is the diffusion length and *D* is the diffusivity of the species, the characteristic time for GFP to diffuse 10 μm to the open top of the microwell is ~0.6 s. On the other hand, the delivery of reagents over short distances is efficient. Using similar scaling, we estimate the time required for diffusive transport of lysis buffer components (*D*=80 μm^2^ s^−1^ for Triton X-100 micelles^34^) from the top of a microwell to the cell to be ~0.7 s.

Consequently, we sought to design the (sc)^2^WB microdevice to diffusively control reagent transport and allow the following: (i) selective lysis and protein solubilization of each cellular compartment using diffusion-based delivery of the DDF buffer pairs and (ii) rapid, near lossless transition to PAGE of both the 14 pL cytoplasmic and 2 pL nuclear compartments (Figures 1c and d). To afford both capabilities, we arrived at a multilayer device design consisting of a thin hydrogel ‘base’ layer (40 μm thick, on a microscope slide) stippled with cell-isolating microwells that is capped by thick buffer-soaked hydrogel ‘lid’ layers (500 μm thick).

First, to afford the rapid, sequential delivery of the DDF buffers, a sequence of the buffer-soaked hydrogel ‘lids’ is used. Once in direct fluidic contact, the lids diffusively deliver lysis buffer constituents into the microwells. An essential aspect of the lid design is the elimination of the convective delivery of reagents (pouring) because we have observed ~40% lysate loss when whole-cell lysis buffer is gently poured over the array^25^. Each buffer-soaked lid is 100× the volume of the base layer to approximate an infinite buffer source (that is, the lid volume is orders of magnitude greater than the base layer volume). The 500 μm thick (15%T polyacrylamide (PA) gel) lids are compliant and conform readily to the planar base layer. The lids remain hydrated over the duration of the assay due to their relatively large volume (~10× greater than the bottom layer) and the rapid (⩽35 s) electrophoresis steps. Furthermore, the lids synchronize the delivery of each DDF lysis buffer to the microwells and allow the serial application of the two buffers by simply exchanging the lid containing the cytoplasm-specific buffer for the lid containing the nucleus-specific buffer. Second, we directly molded the microwells into the PAGE sieving matrix, providing nearly instantaneous switching from lysis to PAGE by simply applying an electric field across the entire base layer.

### Establishing the orthogonality of the microfluidic DDF system

We sought to empirically validate the orthogonality of the cytoplasmic and nuclear DDF lysis. The cytoplasm-specific lysis buffer comprises non-ionic detergents (digitonin, Triton X-100), which present a bulky head group that solubilizes the cell membrane but do not disrupt the nuclear lamina structure (formed by protein–protein interactions), thus leaving the nucleus intact^13,35^. The nucleus-specific lysis buffer comprises anionic detergents (SDS, sodium deoxycholate) that disrupt the nucleus and solubilize nuclear proteins^13,35^. All detergents are above their critical micelle concentration for effective protein solubilization^35^. To ensure sufficient electrophoretic mobility, both DDF lysis buffers are buffered to pH 8.3 with Tris-glycine^25^ (Supplementary Table S2). Because most cytoplasmic proteins have an isoelectric point below pH 8.3, the Tris-glycine-buffered cytoplasm-specific lysis buffer confers a negative charge and thus an electrophoretic mobility toward the anode^36^. Due to native conditions, the cytoplasmic electrophoretic separation occurs on the basis of both the shape and charge of the proteins^37^. Interestingly, the mild, non-ionic detergents retain protein–protein interactions, as suggested through our observations of an intact lamina structure (Figure 1e) and as corroborated by observations from other groups^13,35^. Such observations suggest that the (sc)^2^WB may be optimized to specifically interrogate cytoplasmic protein–protein interactions and potentially enzymatic activity^35^.

To first empirically validate the cytoplasm-specific DDF buffer, we settled human glioblastoma cells (U373) expressing TurboGFP into the microwell array. To concurrently visualize the cytoplasm (GFP, green) and nucleus of each cell, we stained the DNA with Hoechst 33342 (blue). We applied the cytoplasm-specific DDF buffer lid to the array in <1 s (Figure 1e). During and after the application of the cytoplasm-specific DDF lid, we monitored the solubilization and electrophoresis of TurboGFP via time-lapse microscopy.

Upon application of the DDF lid, we observed cell lysis within 1 s (*n*=3) with the TurboGFP signal filling the microwell. At 25 s of elapsed lysis time, we applied an electrical potential across the base layer (40 V cm^−1^) and observed synchronized electromigration of TurboGFP out of the microwell (Supplementary Figure S2). To quantify the uniformity of the electromigration, we measured the migration distance of TurboGFP across the array and found a coefficient of variance, CV=6.4% (*n*=187 cells).

To verify that the cytoplasmic indicator protein (TurboGFP) was localized to the PAGE gel region and that the nuclear proteins remained localized to the microwells, we immobilized protein in the base layer via UV activation of the benzophenone groups (45 s) in the gel after 14 s of PAGE^25,26,38^. As expected, we did not observe detectable green signal (GFP) within the microwells (*n*=3, SNR<3). As a proxy for the maintenance of the nuclear structure after cytoplasmic PAGE, we imaged Hoechst-stained DNA and observed retention of the DNA in each microwell (*n*=3). To inspect the state of the nuclear proteins, we immunoprobed for lamin A/C, a nuclear envelope protein (Figure 1e, Stage 1). Using end point fluorescence imaging, we observed the GFP signal localized to the PAGE regions of the gel base layer and lamin A/C signals localized to the microwells (Figure 1e).

After the cytoplasmic PAGE step (Figure 1e, Stage 1), visual inspection of cell-laden microwells revealed both a stained DNA signal and a lamin A/C signal. The positive signals indicate that the non-ionic detergents comprising the cytoplasmic lysis buffer maintained intact nuclei in each microwell, corroborating literature observations^39,40^. On the basis of similar observations from bulk (pooled cell) DDF, we anticipate that the intact nucleus retains most nuclear proteins^13,14,39^. Nevertheless, careful validation of bulk and single-cell DDF western blotting should be conducted when macromolecular targets are <50 kDa, especially when not bound to DNA, as these smaller species may diffuse out of the intact nucleus within minutes, through nuclear pores^41,42^. As the proteins considered here are retained in the intact nuclei, the molecular targets should be sheltered from any advection generated during lid exchange of the cytoplasmic to nuclear DDF buffers. Further, during lid exchange, visual inspection of the intact nuclei in each microwell did not report the loss of nuclei for any cell analyzed in this study.

**Figure 2:**
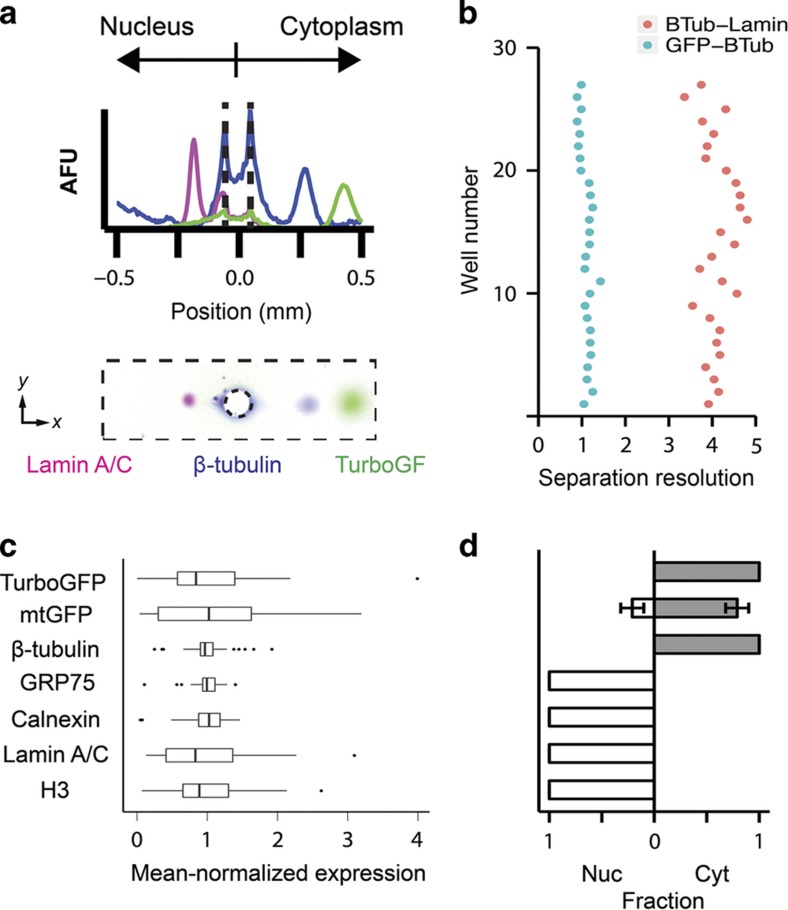
The (sc)^2^WB assay detects a panel of well-described protein targets, thus validating target and localization selectivity. (**a**) Intensity profile and false-color fluorescence for a representative (sc)^2^WB assay (TurboGFP, green signal; β-tubulin, blue signal; lamin A/C, magenta signal; U373-GFP cells; lysis duration: 25 s; PAGE duration: 17 s at *E*=40 V cm^−1^). Dashed lines in the intensity profile denote the microwell border. Cytoplasmic proteins are to the right (west) of the microwell and nuclear proteins are to the left (east). (**b**) Separation resolution of a 1-mm PAGE separation distance (*n*=27 cells). (**c**) Mean-normalized expression (AUC/AUC_mean_) and (**d**) subcellular localization (Nuc=AUC_nuclear_/AUC_total_, Cyt=AUC_cytoplasm_/AUC_total_) as determined by the (sc)^2^WB for membranous organelles: mitochondria-targeted GFP, Calnexin (ER), and GRP-75 (mitochondrial matrix), cytoplasmic (TurboGFP, β-tubulin), and nuclear (lamin A/C, H3) targets. Error bars are±1 s.d. ER, endoplasmic reticulum; GFP, green fluorescent protein; PAGE, polyacrylamide gel electrophoresis; s.d., standard deviation.

We next scrutinized a range of proteins with accepted and well-characterized single-compartment localization (Supplementary Table S4). For targets localized to a single compartment, we report a 100% localization to that specific compartment (for example, 100% cytoplasmic localization with 0% nuclear localization). An important performance metric relevant to assigning the localization fraction is the limit of detection of the (sc)^2^WB in-gel immunoassay, which is ~45 zeptomoles^25^. For the nucleus-specific targets, we observed 100.0±0.0% (*n*=32) of the lamin A/C signal localized to the region ‘west’ of the microwells (Figure 2a). To increase confidence in the nuclear analysis, we also probed for histone 3, a nucleus-specific protein (Figures 2c and d), and observed 100.0±0.0% (*n*=22) of the histone 3 signal in the nuclear fraction. Next, in assessing the cytoplasm-specific protein panel, we observed 100.0±0.0% (*n*=32) of the TurboGFP and 100.0±0.0% (*n*=32) of the β-tubulin localized to the gel region ‘east’ of each microwell. These findings suggest that the microfluidic DDF system successfully performs rapid compartment-specific lysis of single cells, for ~30 of cells concurrently. The buffer formulations and technique provide nuclear selectivity, even during cytoplasmic lysis and PAGE analysis of the cytoplasmic lysate.

In seeking to assess the suitability of the cytoplasm-specific lysis buffer for organelle-associated proteins, we assayed mouse embryonic fibroblasts for mitochondria-targeted EGFP (mtGFP), GRP-75 (mitochondrial matrix protein), and calnexin (an endoplasmic reticulum-localized protein). End point fluorescence imaging reported 79±11% (*n*=103) of the mtGFP in the cytoplasmic fraction, whereas the GRP-75 and calnexin were found entirely in the nuclear fraction (Figures 2c and d). Although conventional DDF western blots using the same cytoplasmic lysis buffer detergent formulation have successfully solubilized and extracted proteins from membranous organelles in mammalian cells^13^, we hypothesize that the short lysis durations of the (sc)^2^WB (10–30 s versus 1800 s with conventional DDF) may not fully solubilize this subset of targets (that is, mitochondria-targeted GFP and endoplasmic reticulum protein)^13^. The timescale of diffusive losses from the microwells limits the maximum duration of the lysis step (Supplementary Figure S1). Design modifications optimized to mitigate diffusive protein losses should extend the maximum attainable lysis duration, with the possibility of fully solubilizing difficult-to-solubilize compartments (for example, mitochondrial and endoplasmic reticulum proteins).

Next, using the protein panel, we assessed the separation resolution and peak capacity of the bi-directional PAGE (that is, TurboGFP, β-tubulin, and lamin A/C from GFP-expressing U373 cells, Figure 2a). The total separation length was 1 mm, with 0.5 mm in the direction ‘east’ and ‘west’ of the indexed microwell. After PAGE and blotting in uniform 8%T gels, we fit Gaussian distributions to the fluorescence signal from each of the immunoprobed protein peaks to extract peak center (*x*) and shape (*σ*, standard deviation (s.d.); where peak width *w*=4*σ*). The separation resolution (*R*_S_) is defined as $R_{S}=\frac{x_{1}-x_{2}}{0.5\left(w_{1}+w_{2}\right)}$, where the subscripts describe each of the adjacent peaks. From each of the 17 s duration PAGE separations, all peak pairs were fully resolved with the separation resolution between lamin A/C and β-tubulin at 4.27±0.53 and between β-tubulin and TurboGFP at 1.14±0.07 (*n*=32, Figure 2b). Thus, in the (sc)^2^WB, the cytoplasmic and nuclear fractions of the proteome are spatially separated. Owing to advances in microscopy, ICC can resolve the localization of proteins with 20–30 nm resolution but requires the use of image processing to correlate localization with subcellular features^43^. By spatially separating the cytoplasmic and nuclear compartments, the (sc)^2^WB eliminates the need for the challenging cell segmentation algorithms used in ICC^10,11^.

For a conservative estimate of peak capacity (*n*_c_=*L*/*w,* where *L* is the length of the separation axis)^44^, we used the widest protein peak and estimated an *n*_c_=10.37±0.5 (*n*=32). This *n*_c_ places the multiplexing capability the (sc)^2^WB on par with state-of-the-art single-cell protein analysis tools. Antibody barcode assays report 11 protein targets per cell^45^ and ICC reports four to five targets per cell^46^. This novel bi-directional PAGE assay reports a true nucleocytoplasmic profile for each cell, with multiplexing demonstrated up to six protein targets spanning both the cytoplasmic and nuclear compartments of one cell (Figure 1f).

### Spliceosome-associated proteins

The link between nucleocytoplasmic distribution of spliceosome proteins and disease is not fully understood. The spliceosome is a large molecular machine (composed of nuclear RNA and protein complexes) that removes introns from transcribed pre-mRNA in eukaryotic cells^47^ and can generate alternate proteins (splicing), a phenomenon of growing interest in cancer therapy^48^. Two important spliceosome proteins are SFPQ (namely PSF) and PTBP1 (Refs. 49, 50); both are thought to promote aggressive cancer phenotypes with SFPQ aberrantly localized to the cytoplasm in Alzheimer’s disease^48,51,52^. Importantly, assaying spliceosome-associated proteins is challenging due to the promiscuity of the proteins involved and the importance of localization to function^49,53^.

**Figure 3:**
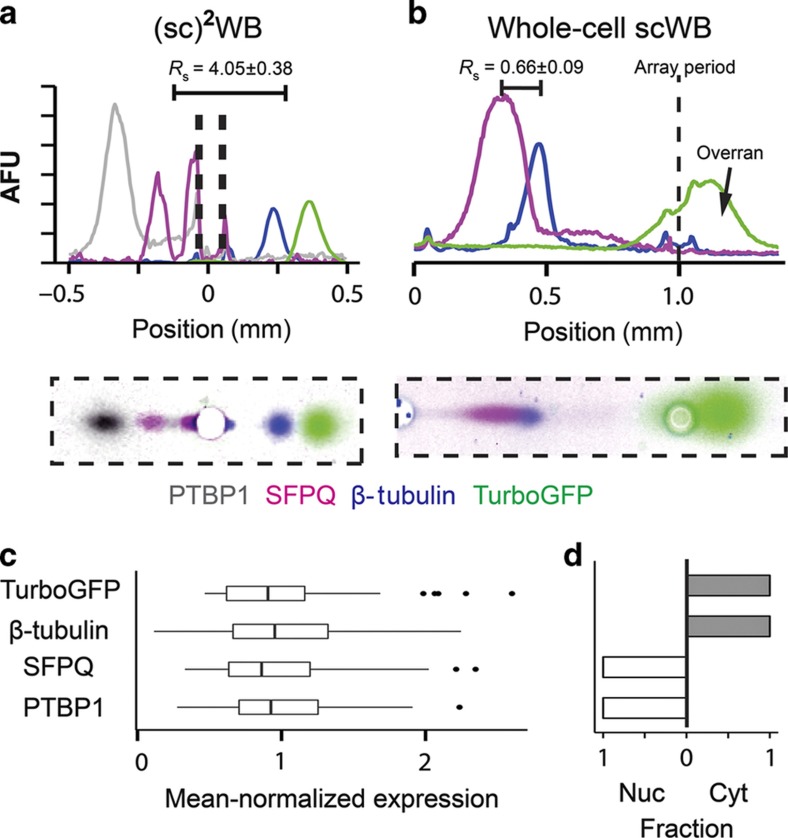
Spliceosome protein localization and expression in single mammalian cells. (**a**) The (sc)^2^WB assigns subcellular localization to cytoplasmic (TurboGFP, green; β-tubulin, blue) and nuclear (SFPQ, magenta; PTBP1, gray) proteins, even when the targets are components of large molecular machines. The dashed line is the microwell border. A representative intensity profile and false-color micrograph are shown here. A dashed line denotes the microwell border in the intensity profile. (**b**) Bi-directional PAGE enhances selectivity because uni-directional PAGE cannot resolve β-tubulin and SFPQ. The dashed line indicates the position of the next row of microwells (array period). Note that in the whole-cell scWB, the TurboGFP band has overrun into the next separation lane. (**c**) Mean-normalized expression (AUC/AUC_mean_) and (**d**) subcellular localization (Nuc=AUC_nuclear_/AUC_total_, Cyt=AUC_cytoplasm_/AUC_total_) of TurboGFP, β-tubulin, SFPQ, and PTBP1 (*n*=44 cells). Error bars are±1 s.d. GFP, green fluorescent protein; PAGE, polyacrylamide gel electrophoresis; s.d., standard deviation.

Here, we sought to measure the expression and localization of four protein targets using the (sc)^2^WB—two cytoplasmic proteins (β-tubulin, 50 kDa, and TurboGFP, 27 kDa) and two nuclear proteins (PTB1, 57 kDa, and SFPQ, 76 kDa; Figure 3a) in GFP-expressing U373 cells. Across an array of 44 cells (Supplementary Figure S3), the multiparameter (sc)^2^WB resolved all four proteins (Supplementary Table S5) with the anticipated compartment localization (Figure 3d). SFPQ had the largest interquartile range (IQR=*Q*_3_−*Q*_1_, where *Q*_1_ and *Q*_3_ are the first and third quartiles, respectively) in mean-normalized expression (IQR_SFPQ_=0.68). Furthermore, the SFPQ and β-tubulin proteins exceeded baseline resolution (*R*_s_=3.33±0.35), even with a small 12.3% molecular mass difference between the pair. To our knowledge, the baseline resolution of two proteins differing by just 7 kDa is the smallest resolved peak pair to date for an immunoprobed single-cell electrophoretic separation of endogenous proteins. Our previously reported whole-cell scWB could not resolve SFPQ and β-tubulin within a ‘uni-directional’ 1 mm PAGE separation distance (Figure 3b), thus underscoring the utility of bi-directional PAGE in subcellular analyses.

### NF-kB protein translocation dynamics

Protein subcellular localization is dynamic. In one established example, stimulation of mammalian cells by LPS found in the outer membrane of Gram-negative bacteria is known to elicit a strong translocation response from the transcription factor family nuclear factor NF-κB^54^. A wide range of downstream processes (for example, cancer progression, inflammation response, and the interplay between innate and adaptive immune systems) are influenced by NF-κB signaling state^55,56^.

**Figure 4:**
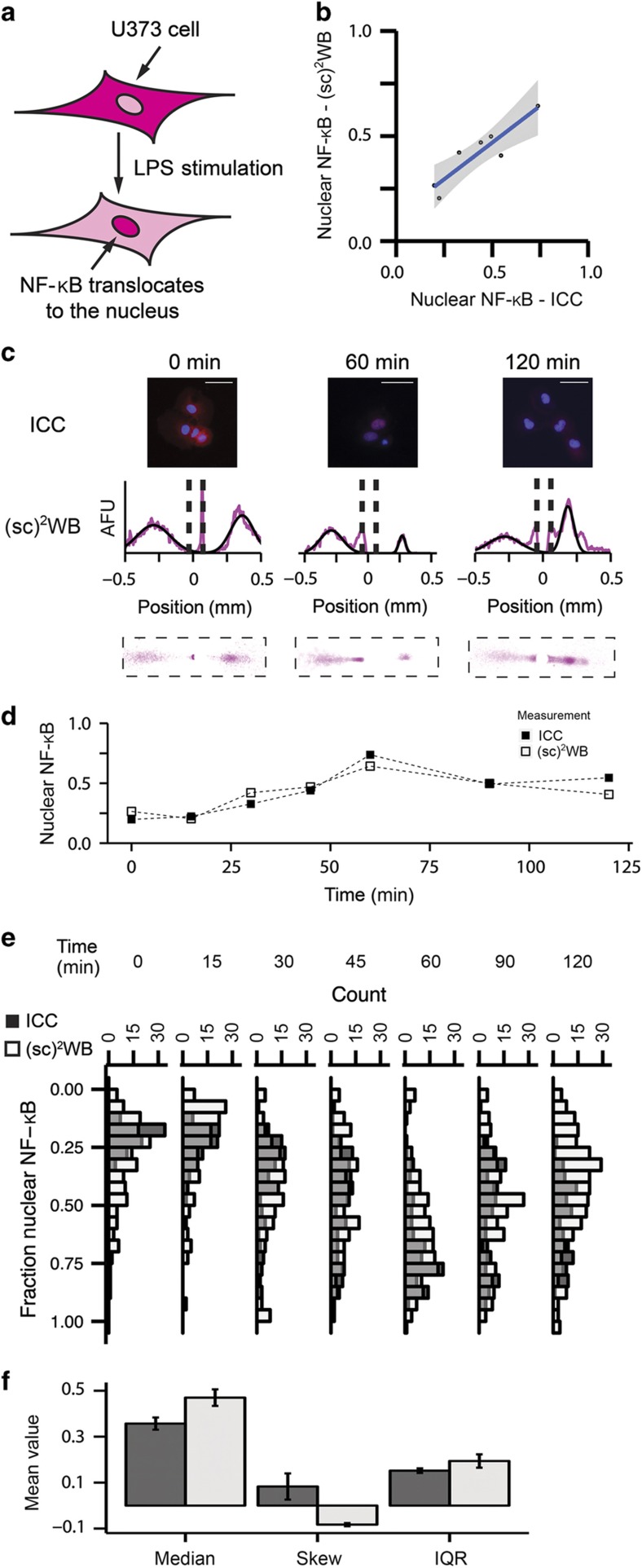
Monitoring dynamic changes in NF-κB localization using the (sc)^2^WB. (**a**) The nucleocytoplasmic distribution of NF-κB changes in response to stimulation with LPS. (**b**) The median localization of NF-κB for each time point as measured by ICC (*x* axis) and the (sc)^2^WB (*y* axis) correlate with *ρ*=0.90. Nuclear NF-κB=AUC_nuc_/AUC_total_. The gray region indicates the 95% confidence interval. (**c**) False-color fluorescence micrographs from ICC and (sc)^2^WB analysis of U373 cells at different times after LPS stimulation (U373 cells, 5 μg mL^−1^ LPS). Magenta traces on the (sc)^2^WB intensity profiles are the raw signal and the black traces are the Gaussian fits. Dashed lines denote the microwell border. (**d**) The median fluorescence signal (AUC) from NF-κB in the nucleus is determined by ICC and (sc)^2^WB, and reports a similar time-to-peak and translocation trend. (**e**) Histograms of nuclear NF-κB expression over the time course by both ICC and (sc)^2^WB. (**f**) Localization distribution parameters from ICC (*n*=4 wells) and (sc)^2^WB (*n*=3 devices). Error bars are±1 s.d. ICC, immunocytochemistry; LPS, lipopolysaccharide; s.d., standard deviation.

To monitor NF-κB signaling during LPS stimulation without artifact-inducing fixation^8,9^, we stimulated naive U373 cells with LPS (5 μg mL^−1^ for 0–120 min) and measured the nucleocytoplasmic distribution of NF-κB in each cell using both the (sc)^2^WB and ICC. We observed an increase in nuclear NF-κB upon stimulation with LPS (Figures 4a and b) with a time-to-peak of 60 min as determined by the (sc)^2^WB and corroborated by gold-standard ICC assays. Furthermore, we calculated the correlation between the median of the NF-κB distribution medians measured by the (sc)^2^WB and ICC and found appreciable correlation at every time point (*ρ*=0.90, Figure 4b). The deviation of the fit line from a slope of 1.0 and a *y*-intercept at the origin is consistent with the skew toward nuclear localization measured by the (sc)^2^WB (Figure 4f). Further investigation of the nuclear skew of the distribution is ongoing. Whereas the dynamics of NF-κB translocation are LPS preparation-dependent, the behavior reported by the (sc)^2^WB agrees with the dynamics reported in the literature (Figures 4c and d)^6^. Using the 6-h (sc)^2^WB assay, we monitored NF-κB translocation in 1247 individual cells, roughly double the number of cells measured using an overnight ICC assay (Figure 4e). Ready scale-up of single-cell protein assays to large populations of cells is important because the measurement throughput establishes statistical confidence by enhancing confidence in distribution estimators.

We next sought to estimate the technical variation in the (sc)^2^WB assay using the NF-κB model system. Establishing sources of variance is particularly difficult in end point single-cell measurements because the same cell cannot be assayed multiple times (that is, no ‘true’ technical replicates). Thus, we performed (sc)^2^WB replicates on cells sampled from the same starting population and compared with ICC. In comparing with ICC we considered three metrics: median, nonparametric skew, and IQR for the (sc)^2^WB-reported NF-κB expression and localization distribution (Figure 4f). These distribution metrics are nonparametric; thus, they do not assume a specific form of the NF-κB expression or localization distribution. In the first metric, the CV of the distribution median indicates how reproducibly the assay reports the center of the expression distribution, with the (sc)^2^WB reporting a value within 3% of ICC.

As a second metric, the nonparametric skew, *S*, provides a measure of the asymmetry of a distribution, where *S*=(*μ*−*ν*)⁄*σ* where *μ* is the mean, *ν* is the median, and *σ* is the s.d. We observed the skew of the NF-kB distribution by (sc)^2^WB to be: (i) more negative than ICC (*S*_ICC_=0.0835±0.0569, *n*=4 wells, *S*_(sc)²WB_=−0.0840±0.077, *n*=3 devices) and (ii) less variable than ICC (CV_ICC_=68.1%, *n*=4 wells, CV_(sc)²WB_=9.14%, *n*=3 devices). A more negative skew (toward the nuclear proteins) suggests predominantly nuclear localization, whereas a more positive skew suggests more cytoplasmic localization. We do not attribute the more nuclear skew to the localization to poor solubilization of the cytoplasmic NF-κB because we did not observe cross-contamination of proteins in the cytosol (that is, TurboGFP and β-tubulin). Thus, the (sc)^2^WB may reduce technical variance by spatially separating the subcellular fractions, eliminating the need for fixation and separating out confounding background signals. Furthermore, the (sc)^2^WB may enhance access to nuclear proteins (Figure 4f), perhaps owing to a reduction in fixation-induced artifacts^8^. In ICC, observed localization is dependent on both the fixation method^57^ (that is, concentration and choice of fixative) and permeabilization method^8^. In addition, fixation is known to dehydrate cellular proteins and impart structural alternations^58,59^. Further, antibody cross-reactivity leads to spurious localization^9^. The (sc)^2^WB circumvents localization artifacts caused by the fixation and permeabilization in ICC by spatially separating the subcellular compartments through DDF and PAGE, thus providing a higher-fidelity measurement of the nucleocytoplasmic distribution of proteins in single cells.

Comparing the IQR of the ICC and (sc)^2^WB compares the measured heterogeneity in the same cell population; with a larger IQR, the cell population is more heterogeneous. The (sc)^2^WB reported an IQR 20% larger than ICC (IQR_(sc)²WB_=19.4% and IQR_ICC_=15.2%). The larger measured IQR in the (sc)^2^WB could be due to the larger number of samples (*n*_(sc)²WB_=1093, *n*_ICC_=676) enabled by the rapid, parallel nature of the assay, allowing the detection of rare events.

Using NF-κB as a model system, (sc)^2^WB measured the dynamic translocation of NF-κB from the cytoplasm to the nucleus at 15 min intervals. Furthermore, the (sc)^2^WB exhibited reduced technical variation in the measured distribution while measuring 61.7% more cells than ICC. We hypothesize that the reduced technical variation is due to the elimination of fixation and the electrophoretic separation of our target analyte (NF-κB) from the background signal. By reducing technical variation, improving accessibility to nuclear proteins, and increasing throughput, the (sc)^2^WB enables measurement of smaller differences in the nucleocytoplasmic distribution of proteins in single cells, a critical parameter in understanding protein signaling. While reducing technical variation, the fractionation strategy implemented in the (sc)^2^WB is optimized to resolve protein localization to the cytoplasmic and nuclear compartments. Future work will refine the lysis buffers to increase the resolution of the fractionation. The increased throughput and reduced technical variance of the (sc)^2^WB enables the detection of more subtle and rare events in heterogeneous cell populations.

## Conclusions

The subcellular localization of proteins can markedly impact cellular function. Although single-cell immunoassays do exist, detection selectivity and throughput remain analytical challenges. The studies detailed here introduce a subcellular resolution western blot assay that confers detection selectivity beyond that of simple immunoassays with a throughput of 10^3^ cells per 6 h assay. Precise microfluidic control of mass transport allows the detection of proteins in the cytoplasm and nucleus in each cell assayed. Immunoblotting and western blots derive detection selectivity enhancements from the integration of a separation stage before the antibody-based detection stage. Here, in the subcellular western blot assay, the two-parameter assay acts to spatially segregate the confounding background signal from the target signal (which has an impact on target multiplexing in ICC) and translates into robust signal analysis using objective peak detection and not complex image segmentation algorithms that require subjective manual input.

Three distinguishing technology contributions make single-cell western blotting with subcellular resolution possible: (1) the development and validation of a pair of orthogonal lysis buffers to differentially lyse the cytoplasm and then the nucleus of each cell while also functioning as an electrophoresis buffer (that is, optimized detergent concentration for rapid solubilization while minimizing conductivity and selected buffering species offering low conductivity and high protein mobility); (2) the design and fabrication of a multilayer device for serial and synchronized, quiescent diffusion-based application of lysis buffers across a large microwell array; and (3) the design and validation of a bi-directional PAGE assay designed to independently measure and spatially segregate target protein expression in the cytoplasmic and then the nuclear fraction. To ensure broad relevance, we validated the tool on a well-characterized panel of protein targets with known subcellular distributions and then applied the tool to analyze a large protein–RNA complex (spliceosome) and a dynamic translocation (NF-κB).

By harnessing the physics available in miniaturized systems, this is (to our knowledge) the first report of protein separations on multiple subcellular compartments of the same single cell. Compared with existing single-cell protein separations, the subcellular western blot assay surpasses peak capacities, resolving power, and throughput. The bi-directional PAGE format enhances both the peak capacity and resolving power with three fully resolved proteins blotted in a 1 mm separation distance including the resolution of a 7 kDa mass difference (β-tubulin and PTBP1; 12% mass difference).

Furthermore, using an electrophoretic separation to spatially segregate the cytoplasmic and nuclear compartments enhances selectivity over single-stage immunoassays, including ICC. Separating the subcellular compartments obviates the need for fixation and image segmentation algorithms, both sources of variance and potentially spurious results. In addition, the electrophoretic protein separation identifies targets by both electrophoretic mobility and immunoaffinity. As hypothesized and supported by the technical variance analysis described here, the (sc)^2^WB appears less sensitive to both off-target background signal and cell fixation conditions (having no fixation in the microfluidic assay) than ICC. In reducing the technical variance and eliminating fixation artifacts, the (sc)^2^WB should more accurately measure the nucleocytoplasmic distribution of proteins in single cells than ICC.

Looking forward, we envision integrating the phenotypic characterization of each cell with the end point nucleocytoplasmic protein-profiling assays described here. Ongoing research is being conducted on both diversifying and optimizing the DDF lysis buffer chemistries to scrutinize an even wider range of subcellular compartments and organelles. Active settling methods may increase the throughput of the (sc)^2^WB, allowing for the detection of rare cells. Further increasing the subcellular resolution of the (sc)^2^WB will deepen our understanding of how protein translocation drives protein signaling in processes such as cancer metastasis and stem cell differentiation.
