# Supplementary material for: Subcellular western blotting of single cells
Source: Microsyst Nanoeng. 2017 Feb 13;3:16079. doi: 10.1038/micronano.2016.79 (PMC5764185; doi:10.1038/micronano.2016.79)
Supplement: Supplementary Information [file micronano201679-s1.pdf]

Supplementary file

Subcellular western blotting of single cells

Kevin A Yamauchi[1,2] and Amy E Herr[1,2]

Microsystems & Nanoengineering (2017) **3,** 16079; doi:10.1038/micronano.2016.79; Published online: 13 February 2017

Table S1 Abbreviations used in the main text

Abbreviation	Definition
ICC	Immunocytochemistry
IF	Immunofluorescence
DDF	Differential detergent fractionation
(sc)^{2}WB	Subcellular single-cell western blot
PAGE	Polyacrylamide gel electrophoresis

Table S2 Formulation of the cytoplasm- and nucleus-specific lysis buffers

	Cytoplasm-specific buffer	Nucleus-specific buffer
Sodium dodecyl sulfate	—	10 mg mL^{-1}
Sodium deoxycholate	—	5 mg mL^{-1}
Triton X-100	1% v/v	0.1% v/v
Digitonin	0.125 mg mL^{-1}	—
Tris-glycine	0.5x	0.5x

Table S4 Single compartment targets assayed by (sc)^{2}WB

Target	Cell type	Localization
TurboGFP	U373	Cytoplasm
β-tubulin	U373	Cytoplasm[1]
GRP75	mtGFP-expressing mouse embryonic fibroblast	Mitochondria[2]
Calnexin	mtGFP-expressing mouse embryonic fibroblast	Endoplasmic reticulum[3]
mtGFP	mtGFP-expressing mouse embryonic fibroblast	Mitochondria[4]
Lamin A/C	U373	Nucleus[5]
H3	U373	Nucleus[6]

Table S5 Separation resolution (R_S) of adjacent proteins targets in the analysis of spliceosome proteins

Target pair	R_S
TurboGFP, β-tubulin	0.86 ± 0.05
β-tubulin, SFPQ	3.33 ± 0.35
SFPQ, PTBP1	1.09 ± 0.17

Table S3 Lysis and electrophoresis conditions used in (sc)^{2}WB

Targets	Figure	Cell	Cytoplasm			Nucleus		
			t_{lysis} [s]	t_{EP} [s]	E [V/cm]	t_{lysis}	t_{EP}	E [V/cm]
TurboGFP, Lamin A/C	1B	TurboGFP-U373	25	17	40	20	20	40
TurboGFP, β-tubulin, PTBP1, SFPQ, HSP90, Erk	1F	TurboGFP-U373	25	16	40	20	35	40
TurboGFP, β-tubulin, Lamin A/C	2	TurboGFP-U373	25	17	40	20	20	40
mtGFP, GRP75, Calnexin	2	Mouse embryonic fibroblasts	25	8	66.67	20	12	66.67
TurboGFP, H3	2	TurboGFP-U373	25	15	66.67	20	15	66.67
TurboGFP, β-tubulin, PTBP1, SFPQ,	3	TurboGFP-U373	25	16	40	20	35	40
NF-κB	4	U373	25	35	100	25	10	66.67

t_{lysis} is the duration of lysis, t_{EP} is the duration of PAGE, and E is the magnitude of the electric field.

[1]Department of Bioengineering, University of California, Berkeley, CA 94720, USA and [2]The UC Berkeley—UCSF Graduate Program in Bioengineering, University of California, Berkeley, CA 94720, USA.
Correspondence: Kevin A. Yamauchi (kevin.yamauchi@berkeley.edu) or Amy E. Herr (aeh@berkeley.edu)

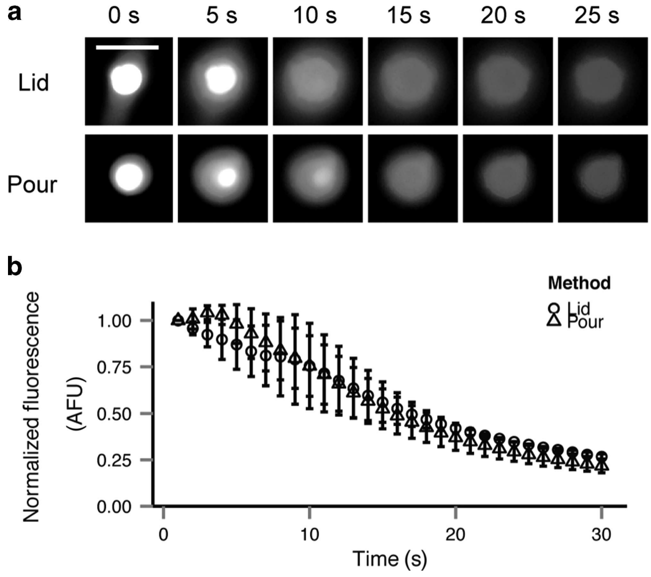

Figure S1 Comparison of diffusive (lid-based) and advective (pour-based) lysis buffer delivery. (**a**) Micrographs of TurboGFP-expressing U373 cells lysing after lid-based and pour-based delivery of the cytoplasm-specific lysis and electrophoresis buffer. (**b**) Normalized fluorescence ($AFU_{current}/AFU_{initial}$) of TurboGFP during lysis of single TurboGFP-expressing U373 cells ($n=3$). After 30 s, only 21.8 ± 3.70% of the TurboGFP remained after pouring buffer over the array and 26.7 ± 1.43% remained with the hydrogel lid delivery. Error bars are ± 1 standard deviation.

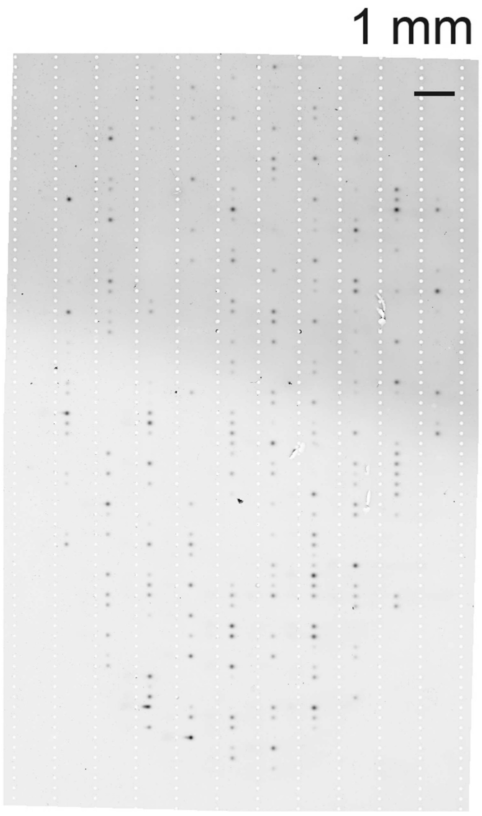

Figure S2 Characterization of electromigration uniformity. Inverted micrograph of TurboGFP-expressing U373 cells assayed with the $(sc)^2$WB for TurboGFP after cytoplasm-specific lysis. TurboGFP-expressing U373 cells were settled into the $(sc)^2$WB device. The cytoplasmic lysis buffer was applied for 25 s. An electric field (40 V cm^{-1}) was applied for 17 s immediately following the completion of lysis. The TurboGFP average peak center location $x = 397.0 \pm 25.4$ μm ($n = 187$), which yields a coefficient of variation, CV = 6.4%.

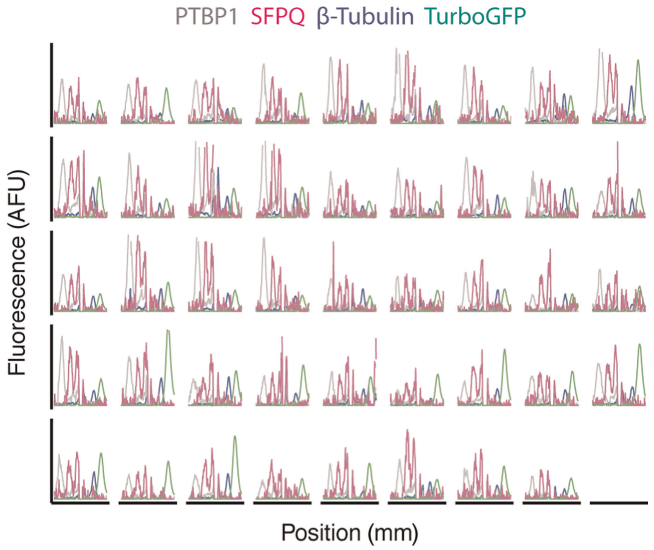

Figure S3 Montage of fluorescence intensity distributions for spliceosome protein targets from concurrent analysis of 44 TurboGFP-expressing U373 cells.

REFERENCES

1 Nogales E, Wolf SG, Downing KH. Structure of the αβ tubulin dimer by electron crystallography. *Nature* 1998; **391**: 199–203.
2 Szabadkai G, Bianchi K, Várnai P, De Stefani D, Wieckowski MR, Cavagna D *et al.* Chaperone-mediated coupling of endoplasmic reticulum and mitochondrial Ca 2 +channels. *The Journal of Cell Biology* 2006; **175**: 901–911.
3 Bergeron JJM, Brenner MB, Thomas DY. Calnexin: A membrane-bound chaperone of the endoplasmic reticulum. *Trends in Biochemical Sciences* 1994; **19**: 124–128.
4 Song Z, Ghochani M, McCaffery JM. Mitofusins and OPA1 mediate sequential steps in mitochondrial membrane fusion. *Molecular Cell Biology* 2009; **20**: 3525–3532.
5 Aebi U, Cohn J, Buhle L, Gerace L. The nuclear lamina is a meshwork of intermediate-type filaments. *Nature* 1986; **323**: 560–564.
6 Simpson RT. Structure of chromatin containing extensively acetylated H3 and H4. *Cell* 1978; **13**: 691–699.
